# Closed atraumatic complete rupture of the flexor halluces longus tendon during forward lunge exercise: A case report

**DOI:** 10.1097/MD.0000000000018409

**Published:** 2019-12-16

**Authors:** Dong Il Chun, Hong Seop Lee, Sung Hun Won, Sang Il Moon, Ki Jin Jung, Jong Hyun Seo, Hyung Ki Cho, Dhong Won Lee, Aeli Ryu, Yudha Manggala, Woo Jong Kim

**Affiliations:** a Department of Orthopaedic Surgery, Soonchunhyang University Hospital Seoul, Yongsan-gu; b Department of Foot and Ankle Surgery, Nowon Eulji Medical Center, Eulji University, Hangeulbiseok-ro, Nowon-gu, Seoul; c Department of Orthopaedic Surgery, Soonchunhyang University Hospital Cheonan, Dongam-gu, Cheonan; d Department of Orthopaedic Surgery, Konkuk University Medical Center, Neungdong-ro, Gwangjin-gu, Seoul; e Department of Obstetrics and Gynecology, Soonchunhyang University Hospital Cheonan, Suncheonhyang 6-gil, Dongam-gu, Cheonan, Korea; f Department of Orthopaedic and Traumatology Surgery, Medicine Faculty, Soegijapranata Catholic University, Semarang, Indonesia.

**Keywords:** closed atraumatic tendon rupture, flexor hallucis longus tendon, foot, forward lunge

## Abstract

**Rationale:**

Acute rupture of the flexor halluces longus (FHL) tendon due to trauma or laceration is a well-known phenomenon. Partial rupture of the FHL tendon caused by tendinitis or stenosing tenosynovitis is common in ballet dancers and athletes. However, atraumatic complete rupture of the FHL is rare: as of 2018, only 7 cases of closed atraumatic complete rupture of the FHL tendon have been reported in the literature. Here, we report on a patient who presented with a closed atraumatic complete rupture of the FHL tendon during a forward lunge exercise.

**Patient concerns:**

A 35-year-old female visited the clinic with pain in the plantar medial aspect of the left foot, along with weakness and loss of great toe flexion. The patient had a normal foot structure and no history of trauma or systemic disease. She performed a forward lunge exercise more than 50 times on 1 leg per day, more than once a week to strengthen her leg muscles. She reported that she felt a slight pain in her left, great toe while exercising for 3 weeks prior to her visit. One week prior to presentation, severe pain occurred suddenly when her left hallux dorsiflexed strongly during an anterior lunge exercise motion.

**Diagnosis:**

Magnetic resonance imaging revealed complete rupture of the FHL tendon near the level of the metatarsal head and neck junction. The lesion was prolonged, with the proximal end displaced to the metatarsal shaft region.

**Interventions:**

Complete rupture of the FHL tendon was treated with a primary suture.

**Outcomes:**

At the 1-year follow-up, active plantar flexion of the interphalangeal joint was possible but joint function had a range of 0° to 25°. Flexion strength was reduced slightly, measuring about 70% when compared to the contralateral side, but flexion strength of the metatarsophalangeal joint was normal.

**Lessons:**

We describe an extremely rare case of complete rupture of the FHL tendon at the level of metatarsal head and neck junction. It should be understood that this injury can occur not only in professional athletes but also in the general public, and we recommend educating personal trainers on how to prevent it.

## 1 Introduction

Acute rupture of the flexor halluces longus (FHL) tendon due to trauma or laceration is well known^[1–4]^ and partial rupture of the FHL tendon caused by tendinitis or stenosing tenosynovitis is common in ballet dancers^[5,6]^ and athletes.^[5,7]^ However, atraumatic complete rupture of the FHL is rare. To our knowledge, Holt was the first to describe a closed atraumatic total rupture of the FHL tendon in 1990; this atraumatic rupture occurred in a 42-year-old female who was a long distance athlete with tenosynovitis in the midfoot. As of 2018, only 7 cases of closed atraumatic complete rupture of the FHL tendon have been reported in the literature.^[8–14]^ Here, we report a patient who presented with a nontraumatic closed rupture of the FHL tendon during a forward lunge exercise.

## 2 Case description

This case report was approved by the Institutional Review Board of Soonchunhyang University Hospital (IRB No. 2019-02-024). The patient gave written informed consent for publication of this report and the accompanying images.

A 35-year-old female visited the clinic with pain in the plantar medial aspect of the left foot associated with weakness and loss of flexion of the great toe. The patient had a normal foot structure and no history of trauma. There were no signs of rheumatoid arthritis, other systemic disease, medication use, or history of steroid or other injections in the affected foot. The patient was not a professional bodybuilder, but about 4 years ago she had begun to perform forward lunge exercises at a fitness center during workouts. She performed a forward lunge exercise more than 50 times per leg per day, more than once a week, to strengthen her leg muscles. She reported feeling a slight pain in her left great toe while exercising for 3 weeks prior to her visit. One week prior to presentation, severe pain occurred suddenly when her left hallux dorsiflexed strongly during an anterior lunge exercise. The pain was worse when walking and standing for a long time, and decreased when resting. The patient visited a local clinic but was diagnosed with no specific findings and was treated with conservative treatment and physical therapy. Her pain continued to worsen and she was transferred to our hospital because a different local clinician suspected she had ruptured her flexor hallucis tendon.

**Figure 1:**
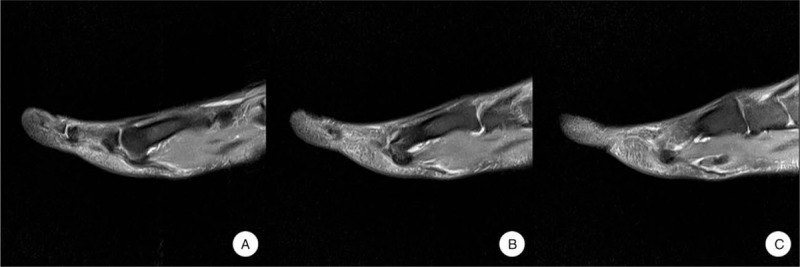
Preoperative magnetic resonance images show that the FHL tendon was completely ruptured at the metatarsal head and neck region and displaced. FHL = flexor halluces longus.

**Figure 2:**
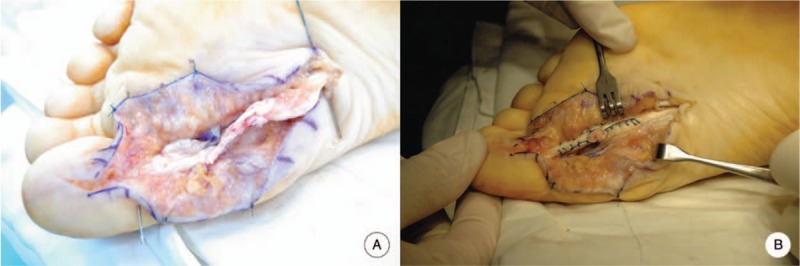
Intraoperative photographs show rupture of the FHL tendon (A) and the tendon after repair using the Modified Becker method (B).

On physical examination, the patient had mild swelling and tenderness on the plantar medial area, about 2 cm proximal to the metatarsophalangeal (MTP) joint, but no palpable mass. Active flexion of the first MTP joint was intact but weakened. There was no active flexion in the interphalangeal (IP) joint of the hallux but a normal active extension of the IP joint. There was no local tenderness at the hallux IP joint. The neurovascular examination was unremarkable. She was diagnosed with a closed atraumatic rupture of the FHL tendon. Plain radiographs of the left foot and ankle revealed no bony deformity of the accessory bone. Magnetic resonance imaging revealed complete rupture of the FHL tendon at the metatarsal head and neck junction. The lesion was prolonged and the proximal end was displaced to the metatarsal shaft area (Fig. 1). Tenosynovitis was detected around the ruptured tendons on either side. This finding was confirmed at surgery. The patient wanted surgery because the pain interfered with her daily life and she wanted to retrieve some movement of the hallux. The authors explained the difference in repair results between a closed rupture and a lacerated rupture of the FHL tendon, and the patient understood this. A plantar incision was made on the hallux metatarsal area to expose the site of the torn FHL tendon. We incised the tendon sheath and noted an interstitial-type rupture of the tendon. The distal end of the ruptured tendon was located at the metatarsal head and neck junction region and the proximal stump was retracted about 1.5 cm, located at the level of the metatarsal shaft area (Fig. 2B). The lesion was treated with a primary suture using a modified Becker method (Fig. 2B). Postoperatively, the foot and ankle were immobilized for 4 weeks in a short-leg cast, in an ankle joint neutral position with the MTP joint flexed plantar by about 20°. The position was changed at the 4-week mark: the ankle joint and MTP joint were both placed in a neutral position and an additional cast fixation was performed for 2 weeks. Immediately after the operation, passive MTP joint flexion exercise was initiated by manual manipulation but active exercise was not allowed. Active plantar flexion and progressive passive dorsiflexion were performed 4 weeks postoperatively. After 2 weeks, the patient was actively exercising with slight resistance. She has only mild, intermittent pain in the medial midfoot 6 months after surgery. She has no discomfort in her daily life and is nearly accomplishing her preoperative level of training. At the 1-year follow-up, active plantar flexion of the IP joint was possible but joint function had a range of 0° to 25°. Flexion strength was reduced slightly to about 70% of that of the contralateral side. However, the flexion strength of the MTP joint was normal. The patient was satisfied with the surgical outcome. The hallux metatarsophalangeal-interphalangeal scale of the American Orthopedic Foot and Ankle Society (AOFAS) was improved from 67 points preoperatively to 90 points postoperatively and the visual analog scale (VAS) for pain was improved from 4 points to 1 point. The ankle activity score improved from 67 to 95 points.

## 3 Discussion

Partial closed rupture of the FHL tendon is known to occur frequently in dancers and athletes.^[5,6]^ However, an isolated closed complete FHL tendon rupture is an unusual injury. Cases without trauma are rarely reported.^[8–14]^

Tendon rupture can be caused by several factors, such as trauma-induced direct injury, repeated microdamage, stress concentration associated with sports, tendon degeneration and abrasions resulting from gliding injury, and underlying diseases such as rheumatoid arthritis.^[9]^

Souza et al^[15]^ described a case of traumatic complete rupture of the FHL tendon and presented a brief overview of the 10 previously published cases of traumatic or atraumatic complete rupture of the FHL. Souza stated that the rupture in his patient was caused by forceful dorsiflexion of the hallux while the tendon and muscle were in plantar flexion, resulting in extreme eccentric loading of the muscle. This mechanism of traumatic complete rupture of the FHL tendon is consistent with the mechanism of rupture reported in other cases.^[2,16,17]^ He emphasized that history is important to fully understand the lesion and to potentially help manage cases of closed complete rupture of the FHL tendon.

Several authors have reported atraumatic complete rupture of the FHL tendon.^[8–14]^ In 2 cases, Thompson et al^[12]^ and Wei et al^[14]^ reported spontaneous closed complete rupture of the FHL tendon under the sustentaculum tali and just distal to the knot of Henry, and the cause of the rupture was not mentioned. Holt and Cross^[8]^ reported a high-performance long-distance runner who suffered from a complete rupture of the FHL tendon midfoot, secondary to an abnormal gait.

Inokuchi and Usami^[10]^ described a case of a closed complete rupture of the FHL tendon at its groove in the posterior process of the talus. They reported that pseudoarthrosis led to abrasion of the FHL tendon, resulting in rupture because the patient continued to play soccer despite his symptoms. Jonbergen et al^[13]^ also reported attrition and rupture caused by osteophytes at the os trigonum.

Romash^[11]^ reported a case of closed rupture of the FHL tendon at the great toe metatarsal head level in a marathon runner who trained regularly. Romash concluded that combined tension and compression of the tendon had compromised blood supply, and that repeated stress over time without adequate healing or recovery time may have contributed to the injury.

Hosokawa et al^[9]^ reported a case of subcutaneous rupture of the FHL tendon at the musculotendinous junction in a soccer player. They assumed that the mechanism of the rupture was abruptly forced dorsiflexion of the hallux while stepping, resulting in stress concentration on the partial fracture site on the groove for FHL tendon, consequently leading to complete rupture.

**Figure 3:**
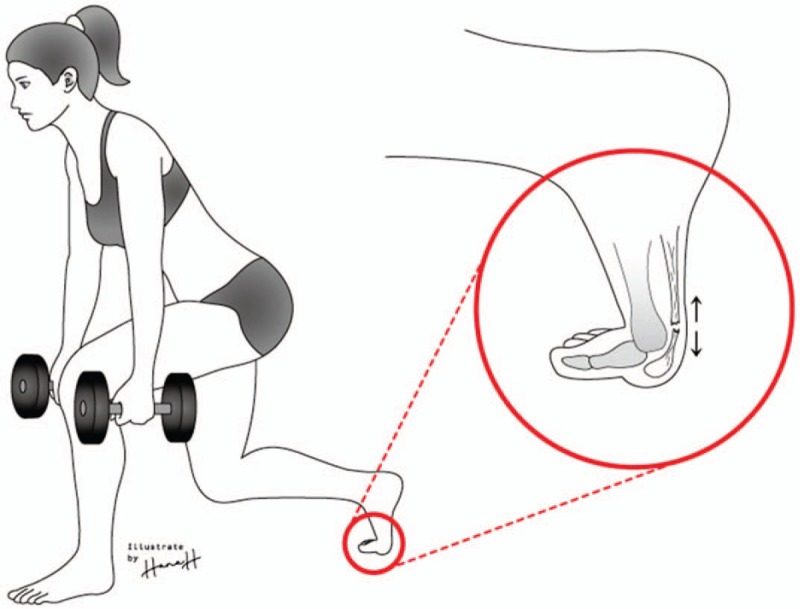
Illustration of the mechanism of the FHL tendon rupture: eccentric contraction of the FHL with the hallux metatarsophalangeal joint in a dorsiflexed position resulted in abrupt stress concentration on the FHL tendon, resulting in complete rupture.

Recently, with improvements in general living standards, many people have become more interested in health and more are participating in exercise for preventive health. A lunge (forward lunge) is an exercise in which 1 foot is advanced forward, the knee is flexed 90°, a static posture is maintained, and the knee joint is returned to the starting posture.^[18]^ When 1 foot is advanced forward, the remaining foot supports the weight, and at this time a strong dorsiflexion force acts on the hallux, which can cause rupture of the FHL tendon (Fig. 3). In our case, the patient experienced some pain while performing lunges, but continued to do so. The pain suddenly increased during lunges 1 week prior to presentation at our hospital. This suggests that tenosynovitis or partial rupture may have occurred before complete rupture.

The FHL tendon progresses from the musculotendinous junction of the posterior-distal aspect of the tibia through the fibro-osseous tunnel at the posterior talus, inferior to calcaneal sustentaculum tali. It maintains a fibrous attachment crossing the flexor digitorum longus at the knot of Henry, which is crossed laterally to medially, remaining deep and along the medial aspect of the foot between the 2 heads of the flexor halluces brevis muscle hallux, and inserting distally on the hallux. According to several previous reports, complete rupture of the FHL tendon may occur at various sites along its entire course: at the insertion site,^[2,17]^ the proximal phalangeal head,^[16]^ the first metatarsal head,^[11,19]^ the knot of Henry,^[1,14]^ the level of talus,^[8,10,12,13]^ and the musculotendinous junction.^[9]^ In our case, the location of the tendon rupture was unique to the metatarsal head and neck junction. Romash^[11]^ noted that the site of subcutaneous rupture is not random but always occurs in one of the lowest resistance points of the tendon.

There have been reports and comments on the treatment of FHL tendon rupture.^[1,2,4,8,10–14,17,20]^ According to Romash,^[11]^ open laceration and closed rupture of the FHL tendon should be distinguished and treated differently, arguing that closed rupture implies failure of a tendon under tension with retraction and disruption of supporting soft tissue. In open lacerations, the cut of the tendon is cleaner and more amenable but a closed tendon rupture is not a clean cut. This difference means that the results may differ.

According to Grispigni et al,^[19]^ tenosuture can allow for repair of active flexion of the great toe only in a few cases, and only partially. They argued that surgical investigation and tenosuture are the most effective methods to alleviate painful symptoms and prevent deformity and hyperextension of the distal phalanx. Hosokawa et al^[9]^ argued that surgical treatment of complete ruptures of the FHL tendon could limit the motion of the hallux IP joint, but could improve the patient's muscle strength while stepping. We agree with this argument.

Surgical treatment of FHL tendon rupture includes tendon suture, tendon transplantation, and tenodesis to the flexor digitorum longus or flexor halluces brevis. The surgical results of closed atraumatic complete rupture of the FHL tendon are as follows:^[8–14]^ primary repair in 3 cases, tenodesis to the flexor digitorum longus tendon in 3 cases, and primary fascial graft in 1 case. In all cases, the clinical results were limited flexion of the IP joint and return of good function.

## 4 Conclusion

We describe an extremely rare case of complete rupture of the FHL tendon at the metatarsal head and neck junction area. A mechanism similar to that which causes rupture in a ballet dancer resulted in a chronic microdamage of the tendon from repeated forward lunge exercises. We can assume this resulted in complete rupture after contributing to a degenerative change or partial rupture of the tendon. In this case, we chose tendon suture. Postoperatively, the patient had limited active flexion of the FHL tendon at the hallux IP joint, and flexion strength reduced by about 70% when compared the contralateral side. However, the flexion power of the hallux was normal and she was satisfied that she could continue her preinjury level of exercise without pain.

It should be understood that this injury can occur not only in professional athletes but also in the general public, and we recommend educating personal trainers on how to prevent it.

## Acknowledgments

The authors thank the Soonchunhyang University Research Fund for support.

## Author contributions

**Conceptualization:** Dong Il Chun, Jong Hyun Seo, Woo Jong Kim.

**Investigation:** Ki Jin Jung.

**Methodology:** Hyung Ki Cho.

**Resources:** Jong Hyun Seo.

**Software:** Sung Hun Won, Aeli Ryu.

**Supervision:** Hong Seop Lee, Dhong Won Lee.

**Visualization:** Sung Hun Won, Sang Il Moon, Yudha Manggala.

**Writing – original draft:** Woo Jong Kim.

**Writing – review & editing:** Woo Jong Kim.

Woo Jong Kim orcid: 0000-0002-4579-1008.
